# Experimental and Theoretical Study of an Actively Q-Switched Tm:YLF Laser with an Acousto-Optic Modulator

**DOI:** 10.3390/molecules26237324

**Published:** 2021-12-02

**Authors:** Lei Guo, Yaling Yang, Ruihua Wang, Baitao Zhang, Tao Li, Shengzhi Zhao, Jingliang He, Kejian Yang

**Affiliations:** 1 School of Mechanics and Photoelectric Physics, Anhui University of Science and Technology, Huainan 232001, China; leiguo@aust.edu.cn (L.G.); yalingyang_opt@163.com (Y.Y.); 2 Institute of Novel Semiconductors, State Key Laboratory of Crystal Materials, Shandong University, Jinan 250100, China; rhwang@sdu.edu.cn (R.W.); btzhang@sdu.edu.cn (B.Z.); jlhe@sdu.edu.cn (J.H.); 3 China Key Laboratory of Laser & Infrared System (Ministry of Education), Shandong Provincial Key Laboratory of Laser Technology and Application, School of Information Science and Engineering, Shandong University, Qingdao 266237, China; litao@sdu.edu.cn (T.L.); shengzhi_zhao@sdu.edu.cn (S.Z.); 4 Collaborative Innovation Center of Light Manipulations and Applications, Shandong Normal University, Jinan 250358, China; 5 Shenzhen Research Institute of Shandong University, Shenzhen 518057, China; 6 State Key Laboratory of Quantum Optics and Quantum Optics Devices, Shanxi University, Taiyuan 030006, China

**Keywords:** actively Q-switched, Tm:YLF laser, rate equation model

## Abstract

We report the characteristics of a diode-end-pumped, high-repetition-rate, acoustic-optic (AO) Q-switched Tm:YLF laser operating from 5 kHz to 10 kHz. In the continuous-wave (CW) regime, a maximum average output power of 8.5 W was obtained with a slope efficiency of 30.7%. Under the AO Q-switching regime, a maximum output power of 7.32 W was obtained at a repetition frequency of 5 kHz with a pulse width of 68 ns and a pulse energy of 1.4 mJ, corresponding to a peak power of 21.5 kW. A time-dependent rate equation model is introduced to theoretically analyze the results obtained in the experiment, in which the cross-relaxation phenomenon, upconversion losses and ground-state depletion are taken into account. Additionally, the evolution processes of population inversion density and intracavity photon number density with time are also presented. The theoretical results well predict the dependence of laser output characteristics of Tm:YLF crystal on the incident pump powers.

## 1. Introduction

Recently, 2-micrometer lasers have attracted much attention in the fields of laser medicine, material processing, laser communication, atmospheric pollution monitoring and mid-infrared optical parametric oscillator. In particular, the high-repetition-rate and short-pulse-width laser source plays an increasingly important role in these fields. As is known to all, the trivalent rare earth ion thulium (Tm^3+^) is a common active ion emitting at 2 μm with the advantages of long fluorescence lifetime and high quantum efficiency, and its pump source is a commercially available laser diode (LD) [1]. Up to now, various Tm^3+^ion-doped crystals (YAP, YAG, LuAG, YLF, etc.) have been widely investigated to achieve a 2-micrometer pulsed laser based on the Q-switching technique [2,3,4]. As a typical representative, Tm:YLF, a natural birefringent crystal with a linearly polarized output, has been used to achieve as high as 87.5 W of CW laser output power at 1907.8 nm [5]. Under the Q-switching regime, Jabczyński et al. reported an acoustic-optic (AO) Q-switched Tm:YLF laser with a pulse width of 20 ns and a pulse energy of 10.5 mJ at a repetition frequency of 10 Hz, corresponding to the peak power of nearly 0.5 MW [6]. In 2015, Korenfeld et al. demonstrated a passively Q-switched diode-pumped Tm:YLF laser based on a Cr:ZnSe saturable absorber with a maximum pulse energy of 4.22 mJ and a pulse duration of 26 ns at the repetition rate of 400 Hz, corresponding to the peak power of 162.3 kW [7]. In 2018, Sheintop et al. demonstrated a high-energy and narrow-bandwidth tunable Tm:YLF laser using a pair of Etalon plates. At a repetition rate of 1 kHz, a pulse energy of 1.97 mJ and a pulse duration of 37 ns were achieved at 1879 nm with a full width at half-maximum (FWHM) of 0.15 nm, corresponding to a peak power of 53.2 kW [8]. However, these studies focus on high-energy lasers and not the research of high-power and high-repetition-rate laser sources.

On the other hand, with the development of the energy level transition mechanism of the Tm^3+^-doped quasi-three-level laser, the rate equation model has been established to analyze laser operating characteristics theoretically, which reveals a good agreement with the experimental results under the CW and passively Q-switched regimes [9,10]. However, there are few numerical simulation studies on actively Q-switched Tm lasers. In the existing models, the laser performances are theoretically investigated by solving the steady-state rate equations to reduce computational complexity, which makes it difficult to simulate the time evolution of photon number and population inversion. Additionally, some key details cannot be presented, such as the pulse build-up time and the evolution of pulse at the initial stage.

In this paper, we demonstrated a high-repetition-rate AO Q-switched Tm:YLF laser. Under CW operation, a maximum average output power of 8.5 W was obtained with a slope efficiency of 30.7%. In the Q-switching regime, a maximum output power of 7.32 W was realized with the shortest pulse width of 68 ns and a maximum pulse energy of 1.4 mJ under a repetition rate of 5 kHz, corresponding to a peak power of 21.5 kW. Besides, we also built a rate equation theoretical model of the diode-end-pumped Tm:YLF laser to theoretically analyze the results obtained in the experiment.

## 2. Experimental Setup

**Figure 1:**
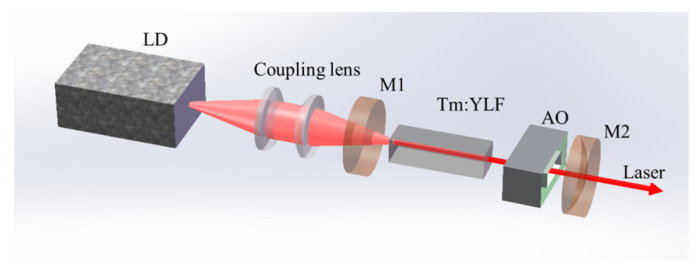
Experimental setup of diode-end-pumped AO Q-switched Tm:YLF laser.

Figure 1 shows the experimental setup of the diode-end-pumped AO Q-switched Tm:YLF laser. The a-cut Tm:YLF crystal had a doping concentration of 3.5 at.% with a dimension of 3 mm × 3 mm × 10 mm. The crystal was wrapped in indium foil and mounted in a copper block cooled by water to 18 °C. Both surfaces were antireflection (AR)-coated from 750 nm to 850 nm and 1800 nm to 2150 nm. A 35 W fiber-coupled LD emitting at 790 nm with a core diameter of 200 μm and a numerical aperture of 0.22 was used as the pump source. The pump light was focused into the laser crystal through a 1:2 imaging module with a spot diameter of 400 μm. The used two-dimensional AO Q-switch (The 26th Electronics Institute of Chinese Ministry of Information Industry) which was made of fused quartz with a physical length of 50 mm and an acoustic aperture of 3 mm × 3 mm, had a diffraction efficiency of 90%. To reduce the insertion losses, both surfaces of the fused quartz were AR-coated at 2 μm. The flat–concave cavity was designed with a length of 80 mm. M1 was a flat input mirror and high reflectivity (HR)-coated from 1850 nm to 2150 nm (reflectivity > 99.5%) and AR-coated from 750 nm to 850 nm (reflectivity < 0.5%). M2 was a plane–concave output mirror (R = −200 mm) with the transmission of 20% from 1850 nm to 2150 nm. The laser pulse trains were recorded by a fast InGaAs photodetector (EOT, ET-5000) with a rise time of 35 ps and monitored by a digital oscilloscope (1 GHZ bandwidth, Tektronix DPO 7102). The average output power was measured by a laser power meter (PM100, Thorlabs).

## 3. Experimental Results

**Figure 2:**
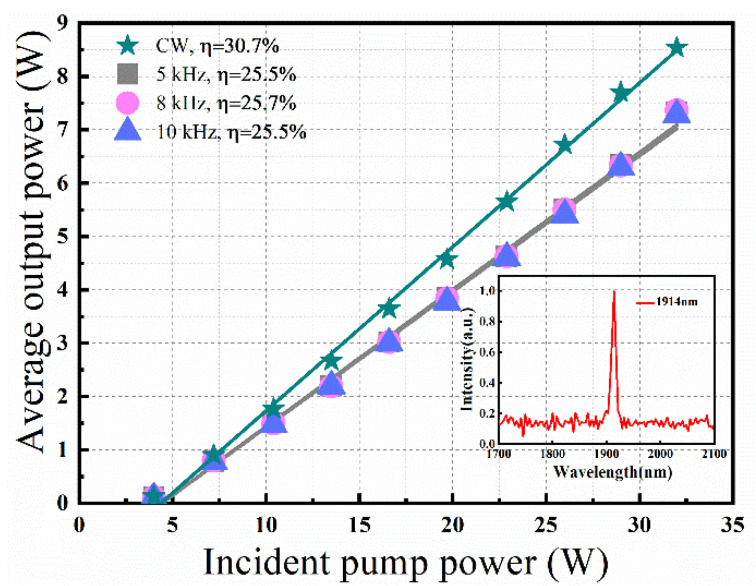
The average output power of diode-pumped CW and AO Q-switched Tm:YLF laser. Inset: output spectrum.

Firstly, the CW running performance of the Tm:YLF laser can be seen in Figure 2. Under the pump power of 32 W, the maximum average output power was 8.5 W for the experiment, corresponding to the slope efficiency of 30.7%.

Subsequently, when the AO Q-switch was turned on, stable high-repetition-rate pulse operation was realized with the repetition frequency varying from 5 kHz to 10 kHz. As shown in Figure 2, similar output powers and slope efficiencies were obtained under different repetition frequencies. Because when the pulse period is much shorter than the upper-level lifetime of the laser crystal, the spontaneous radiation loss can be ignored. Under the incident pump power of 32 W, the maximum average output powers were 7.32 W, 7.36 W and 7.28 W under the repetition rates of 5 kHz, 8 kHz and 10 kHz, corresponding to the slope efficiencies of 25.5%, 25.7%, and 25.5%. In the inset of Figure 2, the output spectrum was recorded by a spectrometer (AvaSpec-NIR-S-350-2080) at a center wavelength around 1914 nm with an FWHM of 7 nm. The ONSR (optical signal to noise ratio) for the obtained laser emission was 34.4:1 based on Gaussian fitting. Considering that the resolution of the spectrometer is only 4 nm, the measured spectral width is not accurate.

**Figure 3:**
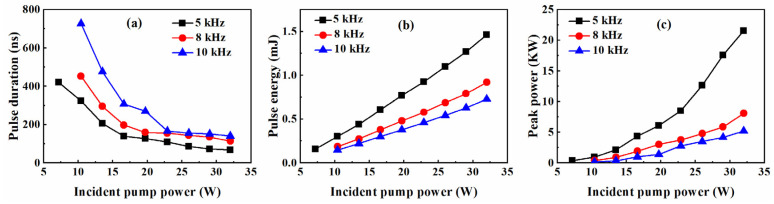
Experiment results for the AO Q-switched Tm:YLF laser: (**a**) pulse durations, (**b**) pulse energies and (**c**) peak powers versus the incident pump powers.

**Figure 4:**
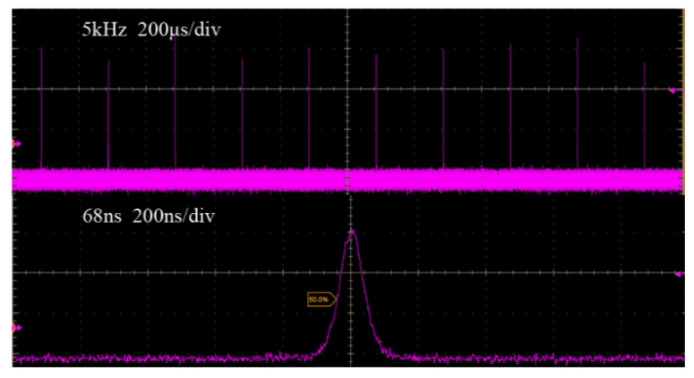
The temporal pulse trains and pulse profile of AO Q-switched Tm:YLF laser at the repetition frequency of 5 kHz.

The dependences of pulse durations, pulse energies and peak powers on incident pump powers at different repetition frequencies are recorded in Figure 3. When the pump power was 32 W, the narrowest pulse durations were 68 ns, 114 ns and 140 ns, and the maximum pulse energies were 1.46 mJ, 0.92 mJ and 0.73 mJ, corresponding to the maximum peak powers of 21.5 kW, 8.1 kW and 5.2 kW for the repetition frequencies of 5 kHz, 8 kHz and 10 kHz, respectively. However, the repetition frequency would be reduced by half of the set frequency of the AO Q-switch if the incident pump power was lower than 10 W for frequencies between 8 kHz and 10 kHz. The small stimulated emission cross-section of Tm:YLF means that a considerable fraction of Tm ions need be exited during laser operation [11]. Therefore, this phenomenon will occur if the pump intensity is too low or the accumulation time of population inversion is too short, which was also verified in theoretical simulation. Under the maximum pump power, the temporal pulse train at a repetition frequency of 5 kHz is shown in Figure 4. The bottom of Figure 4 shows the temporal pulse shape with the shortest pulse duration of 68 ns.

## 4. Theoretical Analysis

**Figure 5:**
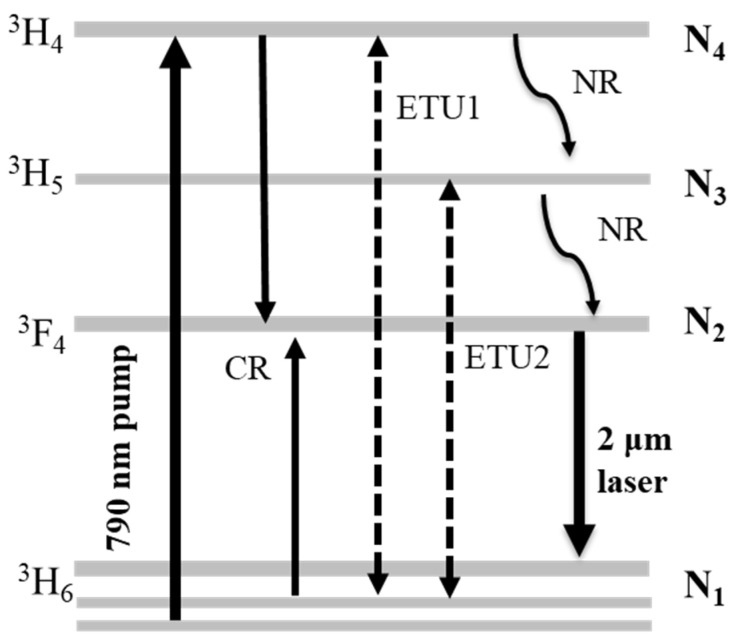
Energy-level transition diagram of Tm^3+^ ion.

To better understand the pulse output characteristics, the quasi-three-level scheme of the Tm^3+^ ion with energy transfer processes is described in Figure 5. Based on 790 nm pump sources, the population of the ^3^H_6_ ground state (N_1_) is excited to the ^3^H_4_ energy level (N_4_). Due to the short lifetimes of the excited states ^3^H_4_ and ^3^H_5_ (N_3_), the population jumps down to the ^3^F_4_ energy level (N_2_) through two non-radiative relaxation (NR) transitions. Finally, the laser emits around 2 μm during the transition from ^3^F_4_ to ^3^H_6_. However, unlike the ordinary quasi-three-level structure, there is a strong cross-relaxation (CR) mechanism between the two Tm^3+^ ions for the ^3^H_4_ + ^3^H_6_ → ^3^F_4_ + ^3^F_4_ level transition processes, which results in a high Stokes efficiency of 0.82, in theory [10].

Then, the coupled rate equation model of an actively Q-switched laser, considering the cross-relaxation phenomenon, upconversion losses and ground-state depletion, is introduced to simulate the characteristics of the emitted pulses as follows Equations (1)–(8):

*dN*_4_/*dt* = *R_p_* − *K_CR_*
*N*_4_*N*_1_ + *K*_*ETU*1_*N*_2_^2^ − *N*_4_/*τ*_4_ (1)

*dN*_3_/*dt* = *K*_*ETU*2_*N*_2_^2^ + *β*_43_*N*_4_/*τ*_4_ − *N*_3_/*τ*_3_ (2)

*dN*_2_/*dt* = 2*K_CR_N*_4_*N*_1_ − 2(*K*_*ETU*1_ + *K*_*ETU*2_)*N*_2_^2^ + *β*_3__2_*N*_3_/*τ*_3_ + *β*_42_*N*_4_/*τ*_4_ − *N*_2_/*τ*_2_ − *σ_e_c*(*ƒ_u_N*_2_ − *ƒ_l_N*_1_)*Φ* (3)

*N*_1_ = *N_Tm_*
− *N*_2_ − *N*_3_ − *N*_4_ (4)

*dΦ*/*dt* = *σ_e_cΦ*(*ƒ_u_N*_2_ − *ƒ_l_N*_1_)*l*/*l*’ − *Φ*/*τ_c_* (5)

*τ_c_* = *t_r_*/*ε* (6)

*t_r_* = 2*l*’/*c* (7)

*ε* = −*ln*(*R*) + *L* + *ζ*(*t*) (8)

where *N_i_* (*i* = 1, 2, 3 and 4) is the population concentration of the Tm level and *N_Tm_* is the Tm ion concentration. *K_CR_* is the cross-relaxation coefficient. *K_ETU*1*_* and *K_ETU*2*_* are the energy transfer upconversion coefficients. The non-radiative lifetime for each level is given by *τ_i_* (*i* = 2, 3, 4), and the branching ratios for levels *m-n* are given by *β_nm_*. *σ_e_* is the stimulated emission cross-section for the 2 μm laser transition. *ƒ_u_* and *ƒ*_l_ are the Boltzmann fractions for the Stark state of ^3^F_4_ and ^3^H_6_ levels. *l*’ = *l_c_* + (*n* − 1)*l* + (*n*_1_ − 1)*l*_1_ is the optical length of the resonator (here, *l* and *n* are the length and the refractive index of the Tm crystal. *l*_1_ and *n*_1_ are the length and the refractive index of the AO crystal). *c* is the speed of light. *Φ* is the photon density in the cavity. *τ_c_* is the cavity lifetime. *t_r_* is the cavity round-trip transit time. ε is the cavity round-trip loss. *R* is the reflectivity of the output mirror. *L* is the loss in cavity. *ζ*(*t*) is the loss introduced during the operation of Q-switch, which is regarded as a step function (0 or *ζ_max_*). *R_p_* is the pumping rate and is given by Equation (9):

*R_p_* = *ηP_in_*/(*πr_p_*^2^*lhν_p_*) (9)

where *P_in_* is the incident pump power, *r_p_* is the pump spot radius, *l* is the crystal length, *h* is the Planck’s constant and *ν_p_* is the pump light frequency. Because of the low stimulated emission cross-section of the Tm:YLF crystal, a considerable fraction of the Tm ions need be excited during laser operation. Therefore, considering the ground-state depletion, the absorption coefficient is reduced according to Equation (10):

*η* = *η*_0_*N*_1_/*N_Tm_* (10)

where *η*_0_ = 80% is the small-signal pump absorption coefficient. The related parameters are shown in Table 1.

**Figure 6:**
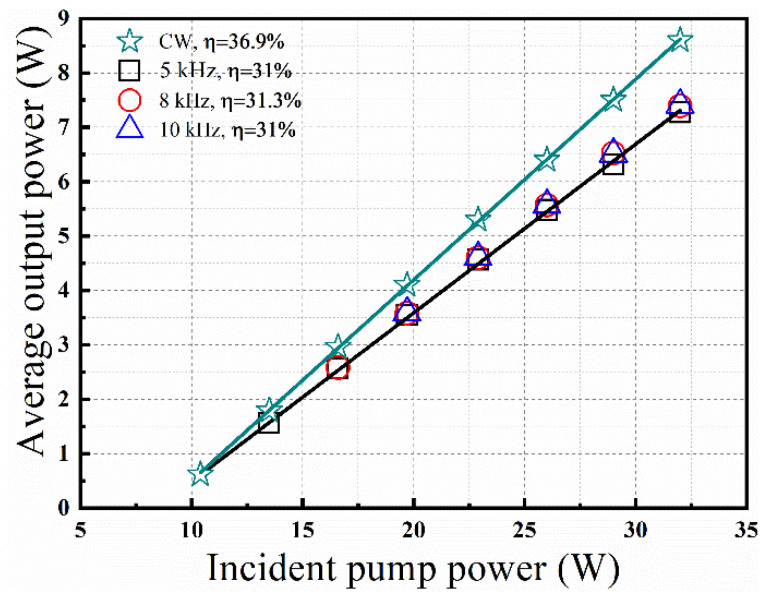
Numerically calculated output power versus the incident pump power for the CW and AO Q-switched Tm:YLF laser.

Firstly, the CW running performance of the Tm:YLF laser is numerically simulated by setting the loss *ζ*(*t*) to 0 over the entire time interval. The rate Equations (1)–(10) are solved numerically by a computer based on the four-order Runge–Kutta algorithm. As shown in Figure 6, the numerically calculated output powers increase almost linearly with incident pump powers. Under the pump power of 32 W, the maximum average output power is 8.6 W for the numerical simulation, corresponding to the slope efficiency of 36.9%. On the whole, the theoretical and experimental results are generally consistent, although the theoretical simulation has higher threshold power.

**Figure 7:**
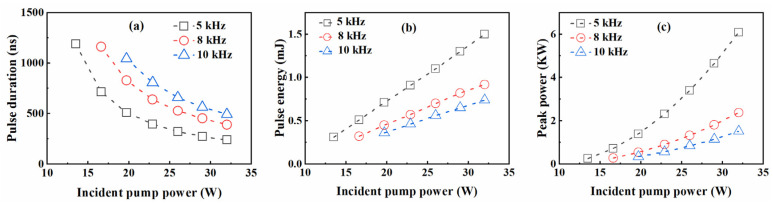
Simulation results for the AO Q-switched Tm:YLF laser: (**a**) pulse durations, (**b**) pulse energies and (**c**) peak powers versus incident pump powers.

Then, in order to predict the pulse characteristics of the Tm:YLF laser, we assume that intracavity photon density changes to 0 instantaneously when the AO switch is turned on, and *ζ*(*t*) is 0 when the AO switch is off. In the numerical simulation, the maximum average output powers are 7.27 W, 7.39 W and 7.41 W with the slope efficiencies of 31%, 31.3% and 31% at 5 kHz, 8 kHz and 10 kHz in Figure 6. The dependences of pulse durations, pulse energies and peak powers on incident pump powers at different repetition frequencies are recorded in Figure 7 for the numerical simulation. When the pump power is 32 W, the experimental and simulated values are as shown in Table 2 under the repetition frequencies of 5 kHz, 8 kHz, and 10 kHz. The simulated pulse energies are consistent with the experimental results, but the experimentally measured pulse widths were about 3~4 times larger than the theoretically simulated values, and the peak powers were about 3~4 times smaller than the simulated values. Considering that the spatial distribution of the beam is ignored in the theoretical simulation, the decrease in pump density has an adverse impact on the pulse width and the peak power compared with the Gaussian transverse profile. In addition, the approximate substitution of some parameters based on the reported literature also has an adverse impact on accurate simulation.

**Figure 8:**
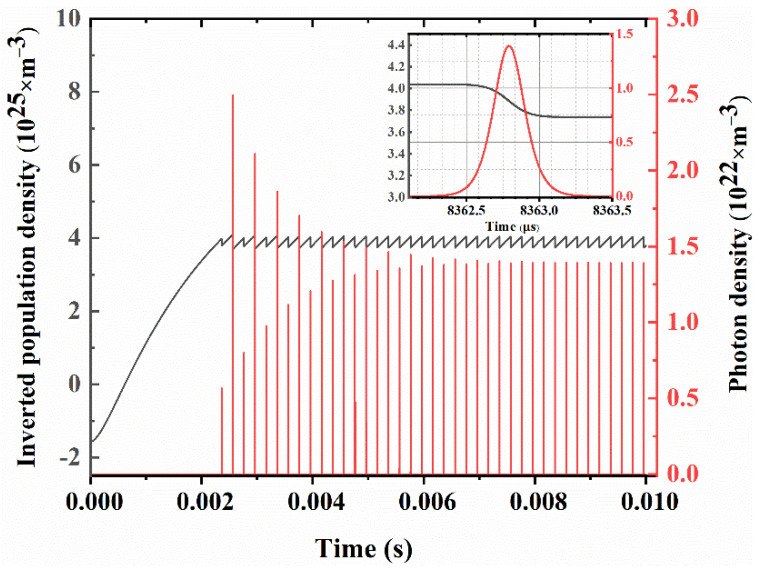
Population inversion density and photon density change in 0.01 s at the repetition frequency of 5 kHz. Inset: expanded picture of single Q-switched pulse.

Figure 8 clearly shows the temporal evolutions of the population inversion density (gray line) and the intracavity photon density (red line) in 0.01 s under the maximum output power of 32 W. As time increases, the population inversion density increases rapidly and reaches saturation near 0.002 s. Then, the laser pulse transitions from an unstable state to a stable state, and the population inversion density also changes with the sawtooth shape. The corresponding expanded picture of the single Q-switched pulse near 0.083 s is shown in the inset of Figure 8.

## 5. Conclusions

In conclusion, a high-repetition-rate AO Q-switched Tm:YLF laser was experimentally studied at repetition rates of 5 kHz, 8 kHz and 10 kHz. Under CW operation, a maximum average output power of 8.5 W was obtained with a slope efficiency of 30.7%. Under the AO Q-switching regime, a maximum output power of 7.32 W was obtained at the repetition rate of 5 kHz with the shortest duration of 68 ns and the maximum pulse energy of 1.4 mJ, corresponding to a maximum peak power of 21.5 kW. In order to further analyze the experimental results, we built a quasi-three-level rate equation theoretical model of an LD-pumped AO Q-switched Tm:YLF laser, and the evolution of the pulse with time was simulated based on the four-order Runge–Kutta algorithm. However, the experimentally measured pulse widths were about 3~4 times larger than the theoretically simulated values, which may be caused by the neglect of the Gaussian distribution of the pump and laser beams and the approximate processing of parameters in the theory. On the whole, this model can effectively predict the dependence of the pulse characteristics on the pump power, especially the average output power and the pulse energy.

## Figures and Tables

**Table 1:** Related parameters used in the numerical model [12,13,14,15,16].

Parameter	Value	Parameter	Value
*h*	6.625 × 10^−34^ J·s	*l_c_*	70 mm
*N_Tm_*	4.89 × 10^20^ cm^−3^	*σ_e_*	3 × 10^−21^ cm^2^
*n*, *n*_1_	1.46, 1.44	*K_CR_*	8 × 10^−18^ cm^3^s^−1^
*ƒ_u_*, *ƒ_l_*	0.2916, 0.0322	*K_ETU*1*_* and *K_ETU*2*_*	1 × 10^−19^ cm^3^s^−1^
*τ_i_* (*i* = 2, 3, 4)	15, 2.258, 0.715 ms	*R*	80%
*β_43_*, *β*_42_, *β*_32_	0.1, 0.03, 0.03	*L*	1.5%
*l*, *l*_1_	10 mm, 5 mm	*A*	0.1256 mm^2^

**Table 2:** Comparison of experiment and simulation results (in brackets).

Repetition Frequencies (kHz)	Pulse Durations (ns)	Pulse Energies (mJ)	Peak Power (kW)
5	68 (239)	1.46 (1.45)	21.5 (6.1)
8	114 (390)	0.92 (0.92)	8.1 (2.4)
10	140 (491)	0.73 (0.74)	5.2 (1.5)

## Data Availability

Data are contained within this article.

## Sample Availability

Samples of the compounds are available from the authors.
